# Constructing Efficient CuO-Based CO Oxidation Catalysts with Large Specific Surface Area Mesoporous CeO_2_ Nanosphere Support

**DOI:** 10.3390/nano14060485

**Published:** 2024-03-07

**Authors:** Yixin Zhang, Fen Zhao, Hui Yang, Siyuan Yin, Cai-E Wu, Tingting Zhou, Jingxin Xu, Leilei Xu, Mindong Chen

**Affiliations:** 1 Collaborative Innovation Centre of the Atmospheric Environment and Equipment Technology, School of Environmental Science and Engineering, Nanjing University of Information Science & Technology, Jiangsu Key Laboratory of Atmospheric Environment Monitoring and Pollution Control, Joint International Research Laboratory of Climate and Environment Change (ILCEC), Nanjing 210044, China; 20211212034@nuist.edu.cn (Y.Z.); 20201248169@nuist.edu.cn (F.Z.); 20211248130@nuist.edu.cn (H.Y.); 20211248096@nuist.edu.cn (S.Y.); 2 College of Light Industry and Food Engineering, Nanjing Forestry University, Nanjing 210037, China; wucaie@njfu.edu.cn; 3 College of Chemical Engineering and Environmental Chemistry, Weifang University, Weifang 261061, China; 20170023@wfu.edu.cn; 4 State Key Laboratory of Low-Carbon Smart Coal-Fired Power Generation and Ultra-Clean Emission, China Energy Science and Technology Research Institute Co., Ltd., Nanjing 210023, China; 20060331@ceic.com; 5 School of Environment and Energy Engineering, Anhui Jianzhu University, Hefei 230009, China

**Keywords:** mesoporous CeO_2_ nanosphere, large specific surface area, redox property, lattice oxygen, CuO-based catalysts, CO oxidation

## Abstract

CeO_2_ is an outstanding support commonly used for the CuO-based CO oxidation catalysts due to its excellent redox property and oxygen storage–release property. However, the inherently small specific surface area of CeO_2_ support restricts the further enhancement of its catalytic performance. In this work, the novel mesoporous CeO_2_ nanosphere with a large specific surface area (~190.4 m^2^/g) was facilely synthesized by the improved hydrothermal method. The large specific surface area of mesoporous CeO_2_ nanosphere could be successfully maintained even at high temperatures up to 500 °C, exhibiting excellent thermal stability. Then, a series of CuO-based CO oxidation catalysts were prepared with the mesoporous CeO_2_ nanosphere as the support. The large surface area of the mesoporous CeO_2_ nanosphere support could greatly promote the dispersion of CuO active sites. The effects of the CuO loading amount, the calcination temperature, mesostructure, and redox property on the performances of CO oxidation were systematically investigated. It was found that high Cu^+^ concentration and lattice oxygen content in mesoporous CuO/CeO_2_ nanosphere catalysts greatly contributed to enhancing the performances of CO oxidation. Therefore, the present mesoporous CeO_2_ nanosphere with its large specific surface area was considered a promising support for advanced CO oxidation and even other industrial catalysts.

## 1. Introduction

Carbon monoxide (CO) is a kind of toxic pollutant that widely exists in vehicle exhaust and industrial waste gas [1,2]. CO can also cause extreme damage to the human health because CO has a high affinity for hemoglobin, which is well known to cause poisoning at high concentrations. Furthermore, CO acts as a precursor to ground-level ozone, leading to the potential for severe respiratory irritation [3,4,5]. Therefore, the removal of CO has received a lot of public attention. Nowadays, there are many ways to remove CO, such as through electrocatalysis [6], thermocatalysis [5,7], and photocatalysis [8]. The thermally catalytic method of CO oxidation could save energy and does not produce secondary pollution. Considering these advantages, the catalytic oxidation of CO is considered a promising route to eliminate CO [9,10]. Regarding CO oxidation, its catalyst systems can be roughly divided into noble metal catalysts (such as Au [11], Pd [3], Pt [12], Rh [13], Ag [14], etc.) and non-noble metal catalysts (such as ZrO_2_ [15], MnO_2_ [16], CeO_2_ [17], Co_3_O_4_ [18], CuO [7], etc.). Although noble metal catalysts commonly exhibit good catalytic performance, their high prices and limited availability hinder their widespread use [19]. Therefore, more and more attention has been paid to non-noble metal catalysts due to their cost-effective and good resistance to sintering. Among these catalysts, CuO-based catalysts have garnered significant research attention owing to their low price, low toxicity, and abundance [20]. However, CuO-based catalysts are commonly deactivated at high temperatures due to their thermal agglomeration, thus affecting the catalytic activity. Therefore, in order to overcome this drawback, CuO is usually dispersed on different catalytic supports, which significantly improve the low-temperature activity of the catalyst [21].

CeO_2_ is a common rare earth metal oxide used as the support for CuO-based catalysts in oxidation reactions. Loading metal oxides on CeO_2_ can enhance the ability of CeO_2_ to release oxygen and further increase the oxidation performance of the catalyst [22]. CuO-CeO_2_ supported catalysts often exhibit unique activity toward the catalytic oxidation of CO. Thus, the CuO-CeO_2_ catalysts are regarded as promising substitutes for precious metal catalysts due to their cost-effectiveness and exceptional activity [17]. Furthermore, the Cu-CeO_2_ catalyst forms Cu/Ce electron pairs, which make the catalyst more active than conventional copper-based catalysts [23,24,25]. At present, many scholars have devoted lots of effort to preparing CuO-CeO_2_ catalysts by employing different methods for CO oxidation reactions. For instance, Jin et al. [26] prepared CuO/CeO_2_ catalysts using different copper salt precursors (Cu(NO_3_)_2_, Cu(CH_3_COO)_2_, Cu(SO_4_)_2_) via the direct solvothermal method. The outcomes revealed that the CuO/CeO_2_ catalyst synthesized with copper acetate as the copper salt precursor displayed the most superior activity for CO oxidation (T_90_ = 78 °C). This exceptional performance could be assigned to the elevated concentration of oxygen vacancies in the catalyst. Gurbani et al. [27] used three methods, the coprecipitation method, the sol–gel method, and the urea–nitrate combustion method, to prepare the CuO-CeO_2_ catalysts for the preferential oxidation reaction of CO. They analyzed the influence of the preparation method on the redox performance and the metal–support interaction between CuO and CeO_2_. They found that the catalyst prepared using the sol–gel method demonstrated superior CO oxidation activity (T_100_ = 177 °C). In contrast, the CuO/CeO_2_ catalyst prepared using the coprecipitation method displayed the lowest catalytic activity. The possible reason for this might be due to the small specific surface area of CeO_2_, which would further limit the dispersion of CuO and accessibility of the reactants to the CuO active sites. Therefore, the reactivity and stability of the supported catalyst could be restricted due to the small specific surface area [17,28]. Consequently, it is crucial to invent a method to prepare CeO_2_ support with a large specific surface area.

Currently, there are many strategies to synthesize mesoporous CeO_2_ according to the recent review by Kumar et al. [29]. The methods used to prepare the large specific surface area CeO_2_ nanomaterials mainly include the soft/hard template method, sol–gel method, etc. [30,31]. Guo et al. [30] prepared a sort of high-surface-area CeO_2_ catalyst for CO oxidation using dodecyl sodium sulfate as the template. The specific surface area of the prepared CeO_2_ could reach 364 m^2^/g after calcination at 300 °C. Furthermore, this catalyst also exhibited good sintering-resistance properties and maintained a large specific surface area of up to 200 m^2^/g when calcined at 600 °C. The large specific surface area of CeO_2_ was mainly derived from the small particle size of CeO_2_ particles, which could provide sufficient defect sites for the activation of the reactants and further enhance the catalytic activity (T_100_ = 300 °C). Gómez et al. [31] used hexadecylamine as the template and cerium acetate as the precursor to prepare the large specific surface area CeO_2_ (161 m^2^/g). The obtained CuO/CeO_2_ catalyst prepared using the impregnation method was used for the selective oxidation of CO. The results showed that the CuO/CeO_2_ catalyst with 6 wt.% CuO loading amount exhibited the best performance (T_100_ = 125 °C). The large specific surface area of the CeO_2_ support not only could reduce the particle size of CeO_2_, but also could facilitate the dispersion of CuO. As a result, the CuO/CeO_2_ catalysts exhibited a strong synergy between the small CeO_2_ particles and the highly dispersed CuO. Luo et al. [32] prepared CuO/CeO_2_ supported catalysts with different loading amounts of CuO using the modified citrate sol–gel method. The heating and calcination were performed in an N_2_ atmosphere during the process of catalyst preparation. The results showed that the citric acid was decomposed into ultrafine carbon powders, which encapsulated the CuO or separated Cu-Ce oxides. The high dispersion of CuO could be obtained on the surface of CeO_2,_ and this catalyst exhibited optimum CO oxidation activity. The CeO_2_ catalyst with 10 mol% CuO content presented the best activity (T_90_ = 100 °C, S_BET_ = 131 m^2^/g).

Although these preparation methods mentioned above could successfully fabricate CeO_2_ supports with large specific surface areas, yet the preparation processes involved were usually very complicated, which required more energy and time consumption [17]. Hence, it is necessary to design and invent a simpler method to prepare structurally stable CeO_2_ supports with large specific surface areas for the CO oxidation reaction with excellent low-temperature performances. The innovation of this work is to use the solvothermal method to prepare CeO_2_ nanospheres with large specific surface areas by adding reactants that are different from other works and controlling the reaction time. Based on the simplicity of the preparation method, the oxidation temperature of CO on the catalyst should be reduced as much as possible.

In this work, a sort of novel mesoporous CeO_2_ nanosphere with a large specific surface area and excellent thermal stability was facilely synthesized by the improved solvothermal method reported elsewhere [33,34]. Then, a series of CuO-based supported catalysts were prepared using the incipient wet impregnation method with the CeO_2_ nanosphere as the support for low-temperature CO oxidation. The effects of the CuO loading amount, the calcination temperature, mesostructure, and redox property on the performances of CO oxidation were systematically investigated. It was found that high Cu^+^ concentration and lattice oxygen content in mesoporous CuO/CeO_2_ nanosphere catalysts greatly contributed to enhancing the performance of CO oxidation. The obtained result demonstrated the advantages of the mesoporous CuO/CeO_2_ nanosphere catalyst with a large specific surface area and excellent redox properties. Furthermore, various characterization techniques, such as the XRD, N_2_ physisorption, SEM, XPS, TEM, H_2_-TPR, in situ DRIFTS, etc., were used to investigate the CuO-based catalysts. The relationship between the catalyst structure and catalytic performance was established.

## 2. Materials and Methods

### 2.1. The Synthesis of the Mesoporous CeO_2_ Nanosphere

The mesoporous CeO_2_ nanosphere was synthesized using the improved hydrothermal method according to the procedures reported in the previous literature [33,34]. The detailed preparation procedure of the mesoporous CeO_2_ nanosphere was summarized in Supplementary Materials S1. The reactants in the preparation method used in this work are different from those in the literature [33], and the solvothermal time is different from that in the literature [34]. The finally obtained mesoporous CeO_2_ nanosphere was labeled as the NS-CeO_2_-T, where the NS represented the nanosphere and T represented the calcination temperature. The mesoporous CeO_2_ nanosphere without calcination was labeled as NS-CeO_2_-P, where P represents the precursor.

### 2.2. The Preparation of the CuO/NS-CeO_2_ Supported Catalysts

The CuO-based catalyst with *x* wt.% CuO (*x* wt.% = m_CuO_/(m_CuO_ + m_support_) × 100%) loading amount was synthesized using the incipient impregnation method. The preparation details of the CuO/CeO_2_ catalyst are found in Supplementary Materials S2. The as-prepared catalysts were denoted as *x*CuO/NS-CeO_2_-T, where *x* represented the mass percentage of the CuO and T represented the calcination temperature.

In addition, CuO-based catalysts supported on the commercial CeO_2_ (Huawei Ruike Chemical Co., Ltd., Beijing, China), SiO_2_ (Shanghai Macklin Bio-Chem Co., Ltd., Shanghai, China), and Al_2_O_3_ (Shanghai Macklin Bio-Chem Co., Ltd., China) were also prepared using the incipient impregnation method. The CuO loading amount was kept at 10 wt.% and the calcination temperature was 500 °C. The as-prepared catalysts were denoted as the 10CuO/C-CeO_2_-500, 10CuO/C-SiO_2_-500 and 10CuO/C-Al_2_O_3_-500, where the C represented the commercial supports.

### 2.3. Catalyst Characterizations

The catalytic supports and catalysts were systematically characterized using X-ray powder diffraction (XRD), N_2_ physisorption, scanning electron microscopy (SEM), energy dispersive spectroscopy (EDS), transmission electron microscope (TEM), X-ray photoelectron spectroscopy (XPS), and H_2_ temperature-programmed reduction (H_2_-TPR). The calcination processes of the CuO-based catalyst precursors were further studied using in situ DRIFTS analyses. Detailed information about the characterizations is listed in Supplementary Materials S3.

### 2.4. The Measurements of the Catalytic Performances

The evaluation of the catalytic activity of CO oxidation was conducted on a fixed-bed reactor. The reaction products were detected using the online gas chromatography (GC-7900, Techcomp, Hong Kong, China). The detailed information for these are summarized in Supplementary Materials S4. The normalized reaction rate of the catalyst was also calculated, and the specific calculation process is summarized in Supplementary Materials S5.

## 3. Results and Discussion

### 3.1. Characterizations of the Supports

**Figure 1:**
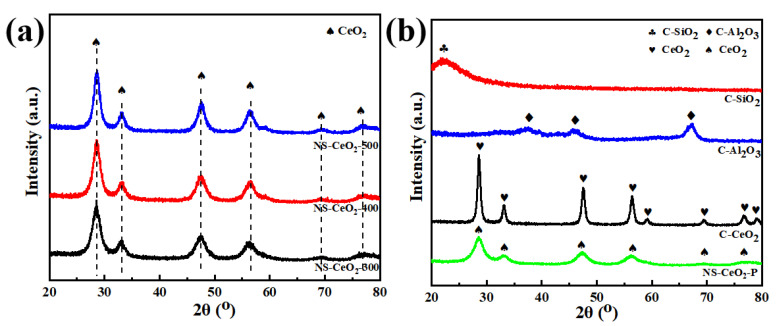
X-ray diffraction patterns of the catalytic supports: (**a**) NS-CeO_2_-T supports; (**b**) NS-CeO_2_-P, C-CeO_2_, C-Al_2_O_3_, and C-SiO_2_ supports.

The XRD characterization was accomplished to determine the crystalline phase state of the mesoporous CeO_2_ nanosphere calcined at different temperatures and the commercial supports. Their XRD patterns are recorded in Figure 1. As presented in Figure 1a, diffraction peaks at 2θ = 28.5°, 33.0°, 47.5°, 56.5°, 69.6°, and 76.7° were observed over all NS-CeO_2_-T supports, which were attributed to the CeO_2_ crystalline phase with a face-centered cubic fluorite structure (PDF #34-0394). Furthermore, as the calcination temperature increased, the intensities of the CeO_2_ diffraction peak were gradually enhanced over the NS-CeO_2_-T supports. The observed phenomenon was attributed to the progressive increase in the grain size of CeO_2_ with the increasing calcination temperature. Similarly, the XRD patterns of the NS-CeO_2_-P commercial supports (CeO_2_, Al_2_O_3_, and SiO_2_) are shown in Figure 1b. Among them, the diffraction peak intensity of the commercial CeO_2_ (PDF #43-1002) support was stronger than that of the mesoporous CeO_2_ nanosphere, which might be attributed to the larger crystalline grain size of commercial CeO_2_. As for the commercial SiO_2_ and Al_2_O_3_ supports, the diffraction peaks of amorphous SiO_2_ (PDF #29-0085) at 2θ = 22.7 ° and the γ-Al_2_O_3_ (PDF #29-0063) at 37.6°, 39.5°, 45.8°, 66.8° could be observed.

**Figure 2:**
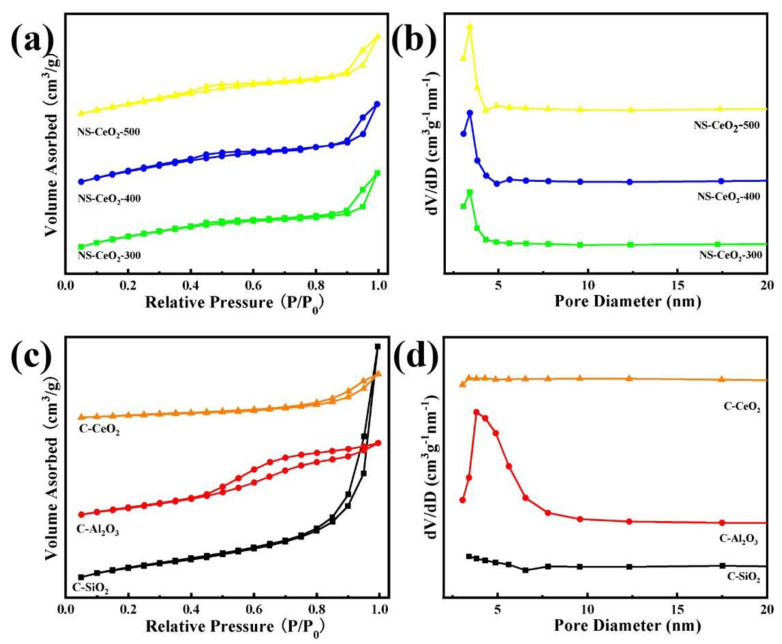
(**a**) N_2_ adsorption–desorption isotherms, and (**b**) pore size distribution curves of the NS-CeO_2_-T supports. (**c**) N_2_ adsorption–desorption isotherms, and (**d**) pore size distribution curves of the C-CeO_2_, C-Al_2_O_3,_ and C-SiO_2_ supports.

In order to analyze the structural properties of the catalytic supports, nitrogen adsorption–desorption analyses of the catalytic supports were carried out. It was found from Figure 2a,c that all samples showed IV-type isotherms. This indicated that the mesoporous structures existed in these investigated supports based on the IUPAC classification [35]. Furthermore, it was worth observing that the NS-CeO_2_-T supports exhibited H3-shaped hysteresis loops, suggesting the existence of narrow-slit-like mesopores [35]. In Figure 2b,d, it was observed that the pore sizes of NS-CeO_2_-T and commercial supports were below 10 nm. The calcination temperature had no effect on the pore size distribution of NS-CeO_2_-T supports. The detailed data regarding the structural properties of these supports are presented in Table 1. Combining the results from Table 1, the specific surface areas of NS-CeO_2_-T supports decreased as the calcination temperature increased. The mesoporous CeO_2_ nanosphere successfully maintained a large specific surface area of up to 181 m^2^/g even after being calcined at 500 °C. This indicated that the current mesoporous CeO_2_ nanosphere exhibited excellent thermal stability at high temperatures. The pore volumes and average pore sizes of NS-CeO_2_-T in Table 1 did not change significantly. Furthermore, the C-CeO_2_ and C-SiO_2_ supports exhibited H3-shaped hysteresis loops, indicating the existence of narrow-slit-like mesopores [35]. The H4-shaped hysteresis loop of commercial Al_2_O_3_ might be due to the existence of slotted pores within the intergranular locations [36].

**Figure 3:**
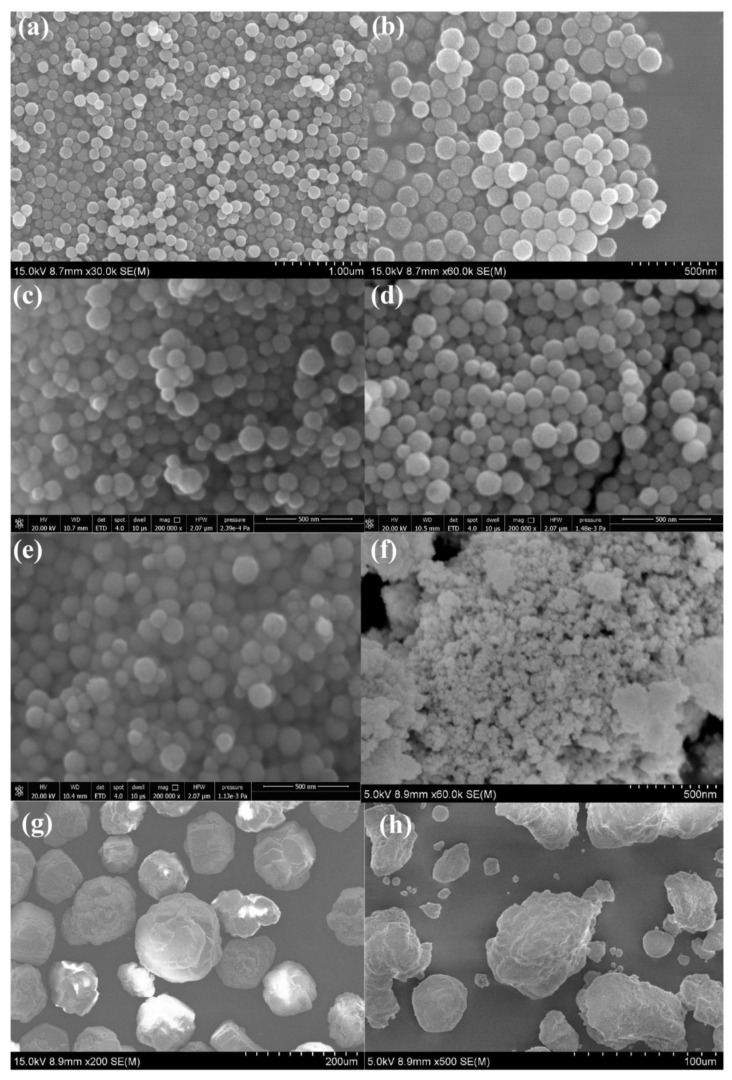
SEM images of the catalytic supports: (**a**,**b**) NS-CeO_2_-P, (**c**) NS-CeO_2_-300, (**d**) NS-CeO_2_-400, (**e**) NS-CeO_2_-500, (**f**) C-CeO_2_, (**g**) C-Al_2_O_3_, and (**h**) C-SiO_2_.

The NS-CeO_2_-T supports calcined at different temperatures and the commercial supports were characterized by SEM to investigate their morphologies and sizes. As shown in Figure 3a,b, the as-prepared CeO_2_ nanosphere without calcination was in perfect spherical shape, with uniform size distribution around 130 nm. In addition, it was found in Figure 3c–e that the uniform spherical morphologies of the NS-CeO_2_-T supports were successfully maintained even after calcination up to 500 °C. This once again illustrated the good thermal stability of these mesoporous CeO_2_ nanosphere materials. In comparison, the reference commercial CeO_2_ support was composed of small CeO_2_ nanoparticles, as shown in Figure 3f. As for the commercial Al_2_O_3_ and SiO_2_ supports, it could be observed from Figure 3g,h that all of them showed irregular morphologies, and their particle sizes were in the micron level.

### 3.2. Characterizations of the as-Prepared Catalysts

#### 3.2.1. In Situ DRIFTS of Calcination Process of CuO-Based Catalyst Precursors

**Figure 4:**
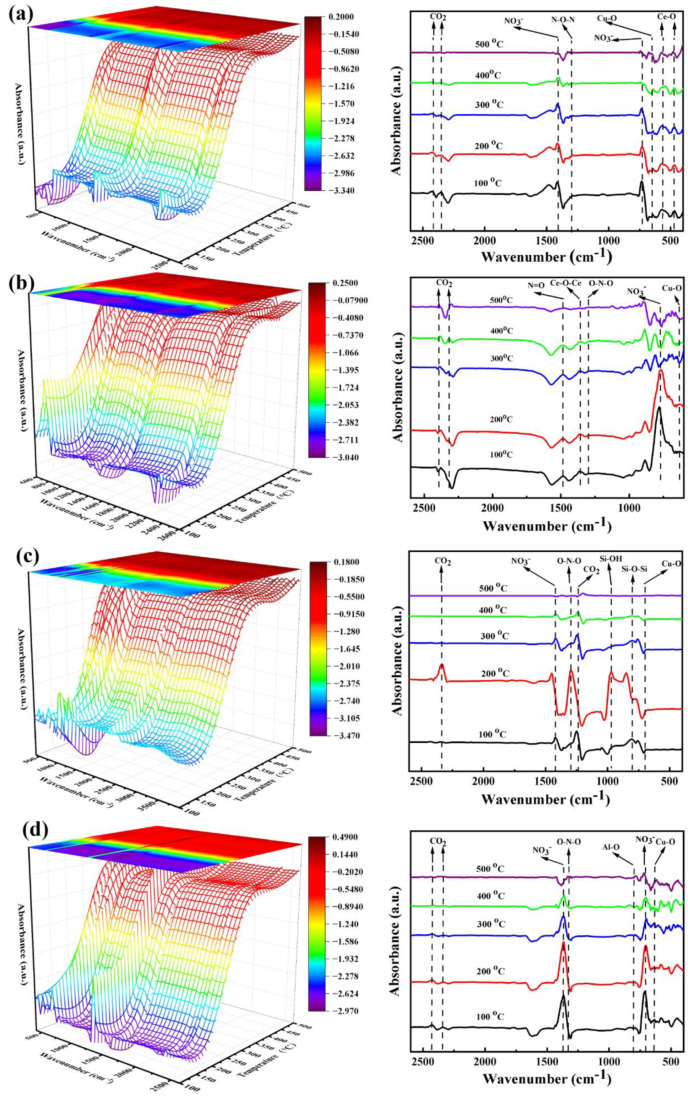
The in situ DRIFTS profiles of the CuO-based catalyst precursors with different supports during the calcination processes: (**a**) 10CuO/NS-CeO_2_, (**b**) 10CuO/C-CeO_2_, (**c**) 10CuO/C-SiO_2_, (**d**) 10CuO/C-Al_2_O_3_.

The in situ DRIFTS analyses were carried out to identify the possible decomposition products of copper nitrate catalyst precursor. It was found in Figure 4 that all catalysts had CO_2_ absorbance peaks (1256 cm^−1^, 2350 cm^−1^) [37,38], which might be due to the adsorption of CO_2_ from the atmosphere on the catalyst surface. Additionally, the infrared absorbance peaks of all catalyst precursors were observed in the range of 1380 cm^−1^ and 760–715 cm^−1^, which were attributed to NO_3_^−^ asymmetric stretching vibration and NO_3_^−^ in-plane bending vibration [37]. Interestingly, the intensity of the NO_3_^−^ vibration peak gradually decreased with the increase in the calcination temperature, suggesting that NO_3_^−^ in the nitrate ligand was gradually decomposed. The calcination of all catalyst precursors revealed the presence of an N=O stretching vibration (1650–1500 cm^−1^) and O-N-O asymmetric stretching vibration absorbance peak (1200–1350 cm^−1^), indicating that NO_3_^−^ had decomposed into NO_2_ gas [39]. Moreover, Cu-O infrared absorption peaks were observed at 632 cm^−1^ [40] for all catalysts, indicating the transformation of Cu^2+^ cation into CuO. The oxide supports themselves also exhibited characteristic absorbance peaks in all catalyst precursors. To be specific, the precursor of the 10CuO/NS-CeO_2_ catalyst (Figure 4a) showed Ce-O peaks at 467 cm^−1^ and 569 cm^−1^ (Ce-O stretching vibration) [41,42,43]. The precursor of the 10CuO/C-CeO_2_ catalyst (Figure 4b) showed an absorbance peak of Ce-O-Ce at 1385 cm^−1^ [44]. The characteristic absorbance peaks of the SiO_2_ support, Si-O-Si symmetric stretching vibration at 805 cm^−1^ and Si-OH stretching at 970 cm^−1^, were observed in the precursor of the 10CuO/C-SiO_2_ catalyst (Figure 4c) [45]. Finally, the precursor of the 10CuO/C-Al_2_O_3_ catalyst (Figure 4d) exhibited a characteristic absorbance peak of Al-O at 765 cm^−1^ (Al-O vibrations in AlO_4_ unit) [46]. The in situ DRIFTS spectra during the calcination process of the catalyst precursors revealed the successful decomposition of the copper nitrate precursor into copper oxide in the presence of air.

#### 3.2.2. XRD Analysis

**Figure 5:**
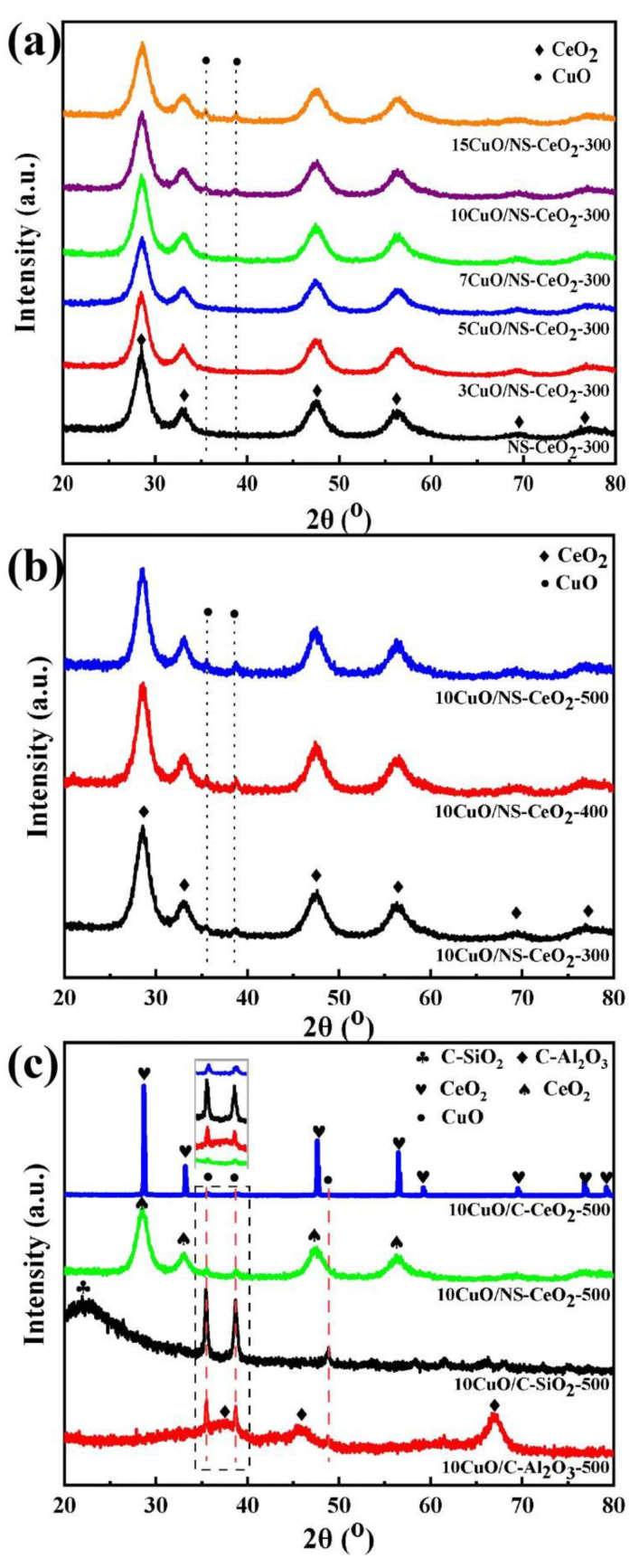
XRD patterns of (**a**) the as-prepared *x*CuO/NS-CeO_2_-300 catalysts; (**b**) the as-prepared 10CuO/NS-CeO_2_-T catalysts; (**c**) the as-prepared 10CuO/C-CeO_2_-500, 10CuO/NS-CeO_2_-500, 10CuO/C-SiO_2_-500, and 10CuO/C-Al_2_O_3_-500 catalysts.

The XRD patterns of the as-prepared *x*CuO/NS-CeO_2_-300 catalysts are displayed in Figure 5a. It was apparent that all these catalysts exhibited diffraction peaks of CeO_2_ (PDF # 34-0394) similar to those of the CeO_2_ support. Meanwhile, the featured diffraction peaks of CuO (PDF # 01-1117) appeared at 2θ = 35.7° and 39.0°. As the content of CuO increased, the intensity of the CuO diffraction peaks gradually increased. Furthermore, the diffraction peaks of CuO could not be detected when the CuO content was below 10 wt.%. The reason for this might be due to the high dispersion of CuO active sites over the NS-CeO_2_ support [47].

Figure 5b exhibits the XRD patterns of 10CuO/NS-CeO_2_ catalysts calcined at different temperatures. The CuO diffraction peaks were observed over these catalysts calcined at three temperatures. Furthermore, it was found that the characteristic diffraction peak of CuO also did not visibly increase, demonstrating the good dispersion of CuO particles on the NS-CeO_2_ support. This suggested that the thermal sintering of CuO was effectively inhibited owing to a powerful metal–support interaction. Similarly, the intensity of the characteristic diffraction peaks of CeO_2_ also did not visibly increase at high calcination temperatures. This indicated no significant increase in CeO_2_ crystalline particles, showing the outstanding thermal stability of the NS-CeO_2_ support.

Figure 5c shows the XRD patterns of the CuO-based catalysts that were prepared using various supports. It was noticeable that all the catalysts exhibited the CuO active site (PDF #01-1117) and their corresponding supports’ (commercial CeO_2_ (PDF #43-1002), NS-CeO_2_ (PDF #34-0394), SiO_2_ (PDF #29-0085), Al_2_O_3_ (PDF #29-0063)) diffraction peaks. However, the diffraction peak intensity of CuO was significantly different over different catalysts with the same CuO loading amount (10 wt.%). It was noteworthy that the CuO diffraction peak intensity over the 10CuO/NS-CeO_2_-500 catalyst was much weaker than that of the 10CuO/C-CeO_2_-500, 10CuO/C-SiO_2_-500, and 10CuO/C-Al_2_O_3_-500 catalysts with commercial catalysts. This indicated that the CeO_2_ nanosphere support was more conducive to the dispersion of CuO.

#### 3.2.3. N_2_ Physisorption Analysis

**Figure 6:**
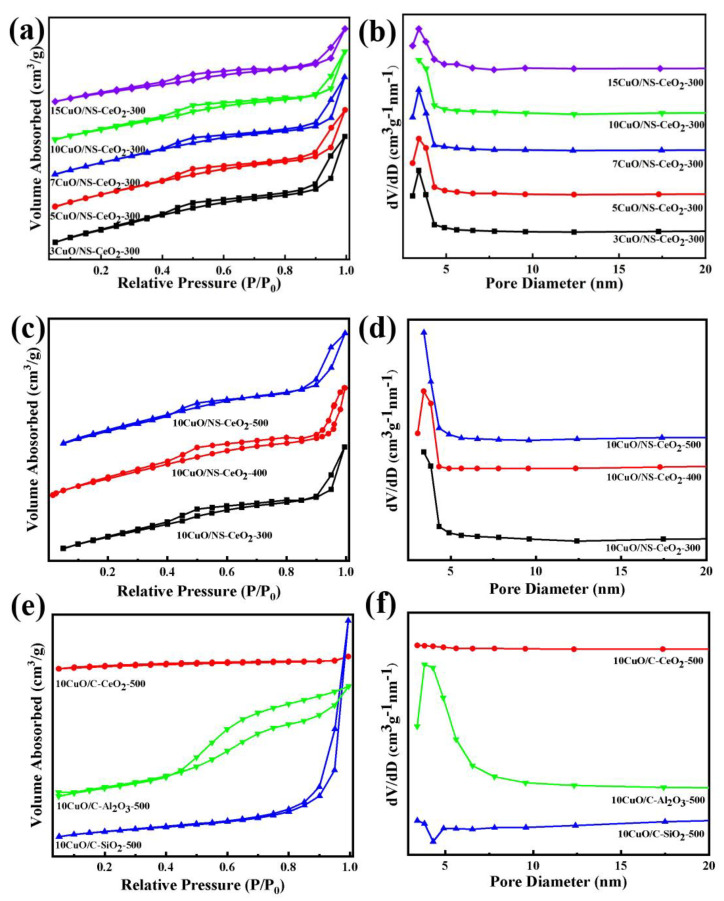
N_2_ adsorption–desorption isotherms and pore size distribution curves of (**a**,**b**) the as-prepared *x*CuO/NS-CeO_2_-300 catalysts; (**c**,**d**) the as-prepared 10CuO/NS-CeO_2_-T catalysts; (**e**,**f**) the as-prepared 10CuO/C-CeO_2_-500, 10CuO/C-Al_2_O_3_-500, and 10CuO/C-SiO_2_-500 catalysts.

Figure 6a depicts the N_2_ adsorption–desorption isotherms of the *x*CuO/NS-CeO_2_-300 catalysts. The type IV isotherms with H3-shaped hysteresis loops are observed in Figure 6a over these catalysts. This indicated that the mesoporous structure of the NS-CeO_2_-300 support was maintained even after loading 15wt.% CuO. Figure 6b displays the pore size distribution curves of the *x*CuO/NS-CeO_2_-300 catalysts. The pore size distribution curves of *x*CuO/NS-CeO_2_-300 catalysts were similar to that of the NS-CeO_2_-300 catalyst, showing that the loading amount of CuO did not affect the structure of the NS-CeO_2_ catalyst. Table 1 displays detailed data regarding the structural characteristics of all the catalysts. As noticed in Figure 6b and Table 1, all these catalysts displayed narrow pore size distribution around 3.4 nm. It was noteworthy that the specific surface area and pore volume of the *x*CuO/NS-CeO_2_-300 catalyst gradually decreased as the CuO content increased, which resulted from a blockage of the mesoporous channels of the NS-CeO_2_ support [47].

Figure 6c shows the N_2_ adsorption–desorption isotherms of the 10CuO/NS-CeO_2_-T catalysts calcined at different temperatures. The IV-type isotherms and H3-type hysteresis loops of the catalysts were maintained after high-temperature calcination. This indicated that neither the calcination temperatures nor the loading of CuO affected the mesoporous structure of the catalysts. This also indicated that the mesoporous structure of NS-CeO_2_ supports was successfully kept after the calcination of catalyst precursors at 500 °C. Figure 6d presents the pore size distribution curves of the 10CuO/NS-CeO_2_-T catalysts. The pore size distribution curves of the 10CuO/NS-CeO_2_-T catalysts were largely similar. Furthermore, the specific surface areas and pore volumes of the as-prepared 10CuO/NS-CeO_2_-T catalysts decreased slightly after calcination at high temperatures. This was mainly attributed to the partial blockage of the mesopores by the nano-sized CuO active sites. However, the 10CuO/NS-CeO_2_-500 catalyst calcined at 500 °C still possessed a high specific surface area up to 176.0 m^2^/g. This demonstrated the excellent structural stability of the 10CuO/NS-CeO_2_ catalysts. In regards to the average pore sizes of these catalysts, they did not change significantly when the calcination temperature of the catalysts increased. This suggested that the mesoporous structure of these catalysts was successfully maintained at high calcination temperatures.

Figure 6e illustrates the N_2_ adsorption–desorption isotherms of the 10CuO/C-CeO_2_, 10CuO/C-Al_2_O_3_, and 10CuO/C-SiO_2_ reference catalysts with commercial supports. The IV-type with an H3-shaped hysteresis loop could be observed in the 10CuO/C-CeO_2_ and 10CuO/C-SiO_2_ catalysts. The IV-type isotherms with an H4-shaped hysteresis loop could be observed in the 10CuO/C-Al_2_O_3_ catalyst. This indicated that the isotherm shapes of C-CeO_2_, C-SiO_2_, and C-Al_2_O_3_ catalyst supports did not undergo significant changes after being loaded with CuO. It was shown in Figure 6f that the pore size distribution curves of these reference catalysts were consistent with their respective supports. The loading of CuO did not change the porous structure of the supports. Similarly, the specific surface areas of the 10CuO/C-CeO_2_ and 10CuO/C-Al_2_O_3_ catalysts in Table 1 were also lower than their respective supports.

#### 3.2.4. SEM, EDS Mapping, and TEM Analyses

**Figure 7:**
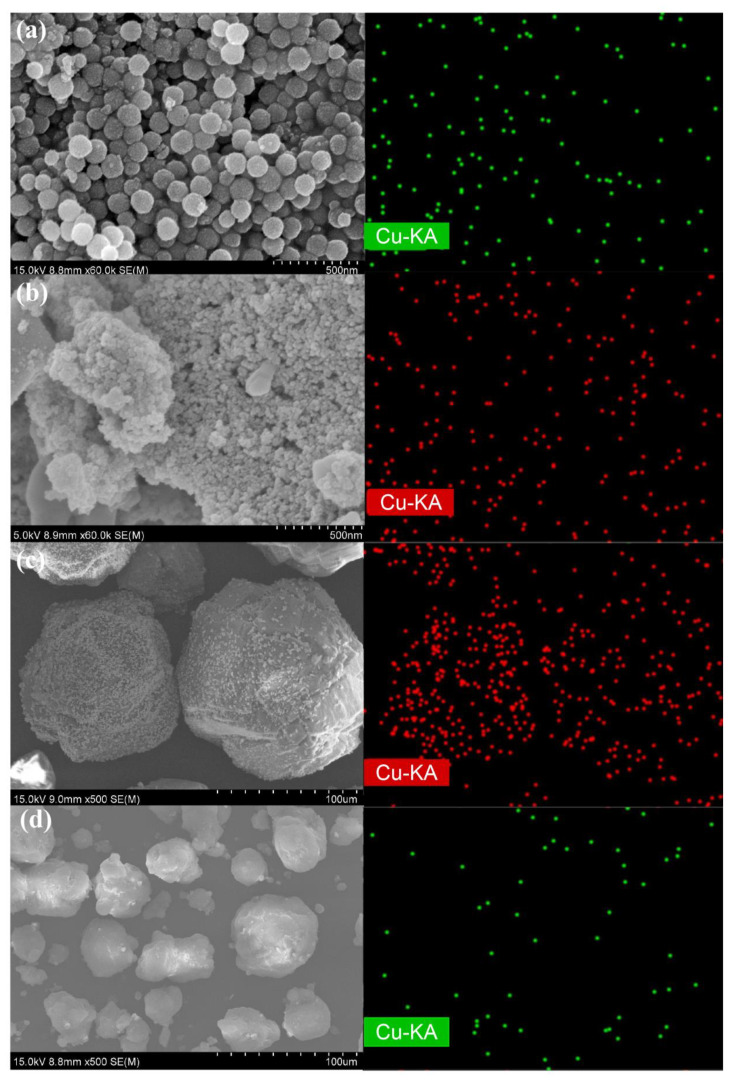
SEM and EDS-mapping images of the as-prepared catalysts: (**a**) 10CuO/NS-CeO_2_-500, (**b**) 10CuO/C-CeO_2_-500, (**c**) 10CuO/C-Al_2_O_3_-500, and (**d**) 10CuO/C-SiO_2_-500 catalysts.

The morphologies and spatial distributions of elements of the as-prepared catalysts were studied using SEM, EDS-mapping, and TEM techniques. The as-prepared 10CuO/NS-CeO_2_-500, 10CuO/C-CeO_2_-500, 10CuO/C-Al_2_O_3_-500, and 10CuO/C-SiO_2_-500 catalysts were selected as the representatives. The SEM and EDS-mapping images are presented in Figure 7. As could be observed, the as-prepared catalysts successfully kept the morphologies of the supports after loading the CuO active sites. Specifically, the mesoporous CeO_2_ nanosphere (NS-CeO_2_) supported catalyst remained with spherical morphology and had a uniform size for the NS-CeO_2_ support after loading the CuO active sites. After loading 10 wt.% CuO and calcining at 500 °C, the 10CuO/CeO_2_-500 catalyst maintained the morphology of nanospheres. The average diameter of the nanospheres was 129.4 nm. It showed that neither loading of CuO nor high-temperature calcination changed the morphology of CeO_2_ nanospheres. This also had been confirmed by the above N_2_ physisorption analyses. Furthermore, the EDS-mapping photos confirmed that the CuO active sites had been successfully loaded on the support with uniform distribution.

**Figure 8:**
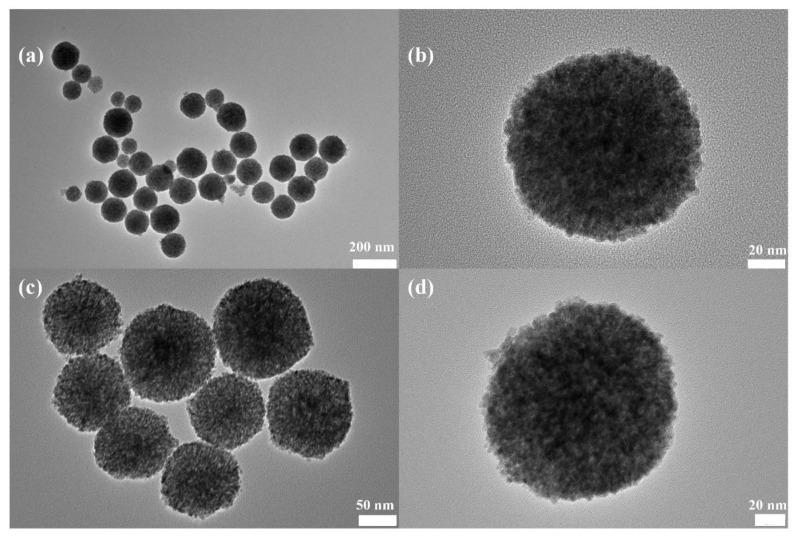
TEM images of the as-prepared catalysts: (**a**,**b**) NS-CeO_2_-P and (**c**,**d**) 10CuO/NS-CeO_2_-500.

Representative catalysts were selected for TEM analysis. The results are shown in Figure 8. The TEM results of the NS-CeO_2_-P carrier are shown in Figure 8a,b. The mesoporous CeO_2_ nanosphere (NS-CeO_2_) supported catalyst appears with mesoporous nanospheres before calcination. The TEM images of the 10CuO/NS-CeO_2_-500 catalyst obtained after loading CuO and calcining at 500 °C are shown in Figure 8c,d. The 10CuO/NS-CeO_2_-500 catalyst still appeared as mesoporous nanospheres, and the sizes of the nanospheres were consistent with the NS-CeO_2_ support. Both the loading of CuO and high-temperature calcination did not affect the morphology of NS-CeO_2_, which is consistent with the results obtained from SEM.

#### 3.2.5. XPS Analysis

**Figure 9:**
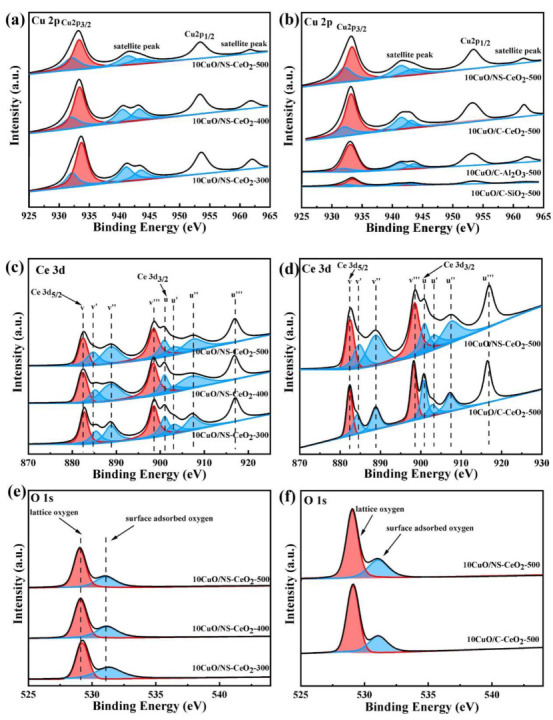
(**a**,**b**) Cu 2p, (**c**,**d**) Ce 3d XPS spectra of the as-prepared 10CuO/NS-CeO_2_-T and the as-prepared 10CuO/C-CeO_2_-500, 10CuO/C-Al_2_O_3_-500, and 10CuO/C-SiO_2_-500 catalysts; (**e**,**f**) O 1s XPS spectra of the as-prepared 10CuO/NS-CeO_2_-T and the as-prepared 10CuO/C-CeO_2_-500 catalysts.

X-ray photoelectron spectroscopy (XPS) was utilized to analyze the surface compositions and chemical states of the elements over the as-prepared catalysts. Figure 9a displays the Cu 2p spectra of the 10CuO/NS-CeO_2_-T catalysts. It was found that these catalysts exhibited two main peaks near 933.2–933.7 eV and 953.3–953.6 eV, which were attributed to Cu 2p_3/2_ and Cu 2p_1/2_ of the Cu^2+^, respectively [48,49]. Furthermore, satellite peaks associated with Cu 2p_3/2_ and Cu 2p_1/2_ were detected in the range of 940.5–943.6 eV and 961.7–962.0 eV, respectively [50]. The satellite peaks were mainly caused by the charge transference based on the pioneer literature [51,52]. It was noteworthy that weak shoulder Cu 2p_3/2_ peaks at 932.0–932.1 eV were also observed. This suggested the existence of low-valence copper oxide species [53]. The previous studies had confirmed that the low-valence copper species mainly existed in the form of Cu^+^ in CuO/CeO_2_ nanocomposites [54]. It was believed that the Cu^+^ species on the catalysts served as the chemisorption and activation sites for the CO molecules during the process of the CO oxidation reaction [55]. Furthermore, the Cu^+^ contents in different catalysts calculated based on the XPS characterization are presented in Table 2. It was discovered that the Cu^+^ content gradually increased with the increase in the calcination temperature. The as-prepared 10CuO/NS-CeO_2_-500 catalyst exhibited the highest Cu^+^ content at 500 °C calcination temperature. Meanwhile, Figure 9b shows the Cu 2p spectra of the as-prepared 10CuO/C-CeO_2_-500, 10CuO/C-SiO_2_-500, and 10CuO/C-Al_2_O_3_-500 reference catalysts. It was found that the peak positions of the Cu 2p spectra over these commercial catalysts were basically consistent with the as-prepared 10CuO/NS-CeO_2_-T catalysts. However, the peak intensities of the Cu 2p XPS spectra over these four catalysts were quite different, although their CuO loading amount was identical (10 wt.%). Therefore, the type of catalytic support had a great influence on the state of the surface CuO. Specifically, the 10CuO/NS-CeO_2_-500 catalyst exhibited the highest surface Cu^+^ content among these catalysts. This suggested that the type of support significantly influenced the Cu^+^ content of the catalyst.

Figure 9c,d depicts the Ce 3d spectra of all the as-prepared catalysts. Generally, the Ce 3d spectra were decomposed into two groups of spin–orbit coupling peaks of Ce 3d_5/2_ (marked as v) and Ce3d_3/2_ (marked as u). Based on the pioneering literature, the v, v’’, and v’’’ peaks belonged to Ce^4+^ 3d_5/2_, while the u, u’’, and u’’’ peaks belonged to Ce^4+^ 3d_3/2_ [56,57]. Additionally, the v’ and u’ peaks represented the characteristic peaks of Ce^3+^ 3d_5/2_ and Ce^3+^ 3d_3/2_, respectively [56,57]. It was noticed in Figure 9c,d that Ce^3+^ and Ce^4+^ coexisted over the surface of all the as-prepared CeO_2_ supported catalysts. As for the as-prepared 10CuO/NS-CeO_2_-T catalysts in Figure 9c, the shapes and positions of their Ce 3d spectra were essentially the same as each other. This indicated that the calcination temperature had minimal impact on the valence state of the Ce species over the catalyst surface. It was widely believed that the formation of Ce^3+^ was caused by the removal of lattice oxygen and the creation of oxygen vacancies [58]. The oxygen vacancy generated by Ce^3+^ would positively promote the reaction (Cu^2+^ + Ce^3+^ ↔ Cu^+^ + Ce^4+^) to produce more Cu^+^, thereby enhancing the catalytic performance of CO oxidation [59]. Furthermore, the specific proportions of Ce^3+^ cations in these catalysts are displayed in Table S1. The as-prepared 10CuO/NS-CeO_2_-500 catalyst had the highest Ce^3+^ proportion. The previous study indicated that the higher Ce^3+^ content promised a greater reduction ability for CuO, which would lead to the formation of more active Cu^+^ sites [60]. The substitution of Cu^+^ cation in the CeO_2_ lattice would result in the generation of additional oxygen vacancies, which greatly enhanced the mobility of lattice oxygen in CeO_2_ [61].

The O 1s spectra of these as-prepared catalysts are shown in Figure 9e,f. It was noticeable from Figure 9e that the shape and position of O 1s spectra of 10CuO/NS-CeO_2_-T catalysts were roughly the same. Generally, O 1s spectra were divided into the main peaks and shoulder peaks. As for the O 1s of the as-prepared 10CuO/NS-CeO_2_-T catalysts in Figure 9e, the main peaks at 529.0–529.2 eV were ascribed to lattice oxygen (O_latt_) [62], and the shoulder peaks at 531.0–531.3 eV were attributed to the surface adsorbed oxygen (O_ads_) species [48]. The content of the surface adsorbed oxygen was proportional to the quantity of surface oxygen vacancy. The lattice oxygen peak area ratios of the as-prepared 10CuO/NS-CeO_2_-T catalysts are listed in Table 3. It was displayed in Table 3 that the content of the adsorbed oxygen decreased from 47.64% to 33.71% when the calcination temperature increased. This denoted that the concentration of the oxygen vacancy in the as-prepared 10CuO/NS-CeO_2_-T catalysts was closely related to the calcination temperature. In addition, it was observed in Table 3 that the lattice oxygen content of the 10CuO/NS-CeO_2_-T catalyst followed the sequence: 10CuO/NS-CeO_2_-500 > 10CuO/NS-CeO_2_-400 > 10CuO/NS-CeO_2_-300. Therefore, the 10CuO/NS-CeO_2_-500 catalyst possessed the largest lattice oxygen content. It was reported that the lattice oxygen of CeO_2_ could react with CO and the O_2_ in gaseous feed gases which subsequently replenished the lattice oxygen consumed [63]. As a comparison, it was found in Figure 9f that the lattice oxygen content of the O 1s spectra of the 10CuO/C-CeO_2_-500 reference catalyst with commercial supports was totally different. These phenomena suggested that the nature of the catalytic support had a significant impact on the amount of lattice oxygen as well.

The specific binding energies of surface elements for all catalysts are presented in Table S2. It was found that the binding energies of Cu 2p_3/2_ were almost similar over almost all these catalysts. Based on the previous analysis, their primary oxidation states or valence states were identified as Cu^2+^, Cu^+^, Ce^4+^, Ce^3+^, and O^2−^. Overall, XPS analysis provided valuable insights into the surface composition and chemical state of catalyst elements, which were used to optimize catalyst design and improve catalytic performance.

#### 3.2.6. H_2_-TPR Analysis

**Figure 10:**
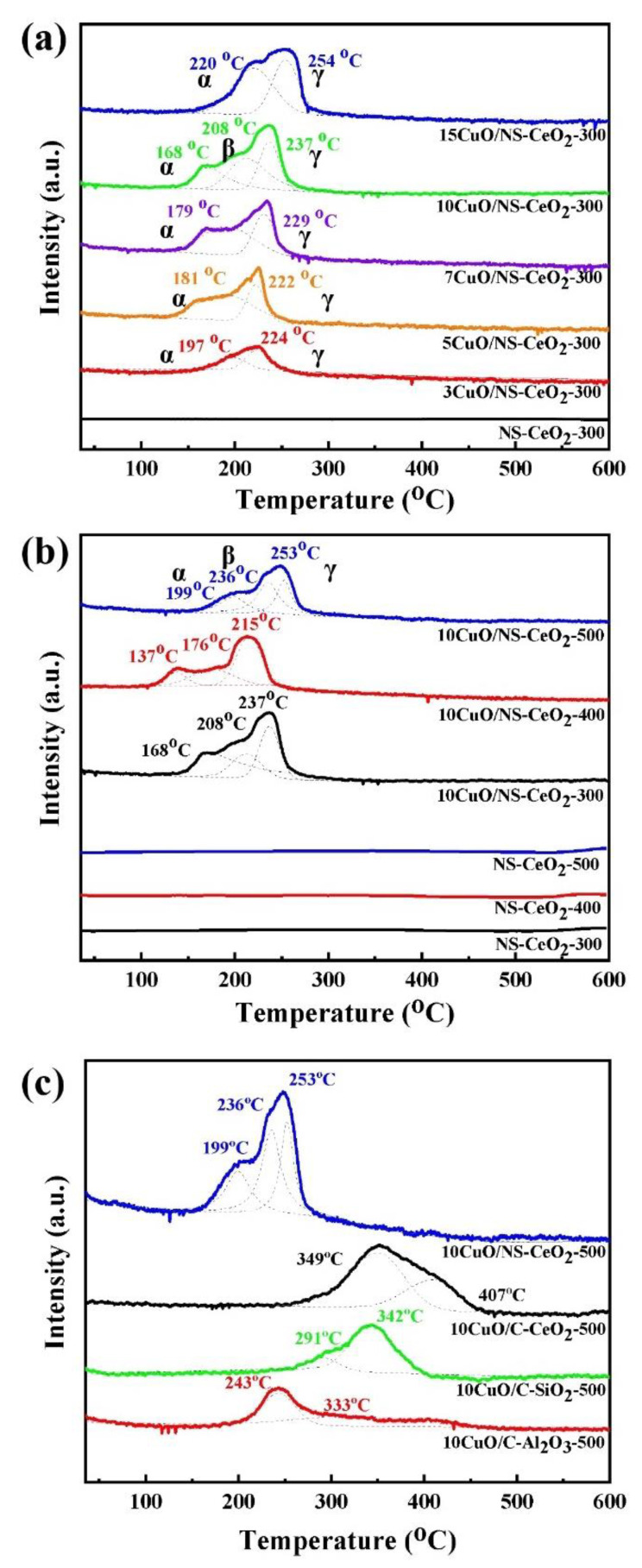
H_2_-TPR profiles of (**a**) the NS-CeO_2_-300 and *x*CuO/NS-CeO_2_-300 catalysts, (**b**) the NS-CeO_2_-T catalysts 10CuO/NS-CeO_2_-T catalysts, and (**c**) the 10CuO/NS-CeO_2_-500, 10CuO/C-CeO_2_-500, 10CuO/C-SiO_2_-500, and 10CuO/C-Al_2_O_3_-500 catalysts.

To examine the interaction between the support and the active metal CuO, H_2_-TPR analysis was conducted over the as-prepared catalysts, and the results in the form of H_2_-TPR profiles are shown in Figure 10. Figure 10a reveals that the NS-CeO_2_-300 support did not show H_2_ consumption between 35 and 600 °C, indicating that CeO_2_ was not easily reduced by H_2_ at lower temperatures. The as-prepared *x*CuO/NS-CeO_2_-300 catalysts displayed two or three reduction peaks. The H_2_ consumption peak of the *x*CuO/NS-CeO_2_-300 catalysts all came from CuO reduction. With the increase in the loading amount of CuO, the reduction peak of the catalyst shifted slightly towards a lower temperature range. The reduction temperature of the catalyst reached the minimum when the loading of CuO was 10 wt.%. Simultaneously, the consumption of H_2_ also increased with the increased loading amount of CuO. The CuO/CeO_2_ supported catalysts typically presented two or three reduction peaks of Cu species at temperatures ranging from 150 °C to 250 °C, namely α, β, and γ reduction peaks according to the previous literature [54,64,65]. Typically, the α reduction peak was ascribed to the reduction in CuO exhibiting strong interactions with CeO_2_ or to the solid solution formed with CuO and CeO_2_; the β reduction peak was assigned to the reduction in highly dispersed CuO species on the surface of CeO_2_; and the γ reduction peak was derived from the reduction in massive CuO species with weak interaction with CeO_2_. This suggested the presence of CuO with strong interactions with CeO_2_ and bulk CuO in the *x*CuO/NS-CeO_2_-300 catalysts. Furthermore, the β reduction peak observed in the 10CuO/NS-CeO_2_-300 catalyst was attributed to the presence of a highly dispersed CuO species after loading 10 wt.% of CuO. Notably, as the loading of CuO increased from 3 wt. % to 15 wt. %, the intensity of the γ reduction peak gradually increased. This denoted the formation of more bulk CuO species. The quantitative ratios of H_2_ consumption are shown in Table 4.

Figure 10b displays the H_2_-TPR profiles of the NS-CeO_2_-T and 10CuO/NS-CeO_2_-T catalysts calcined at varying temperatures. The NS-CeO_2_-T supports did not exhibit noticeable H_2_ consumption. It was noticed that the 10CuO/NS-CeO_2_-T catalysts exhibited similar hydrogen consumption profiles with three reduction peaks from 100 °C to 300 °C. The 10CuO/NS-CeO_2_-400 catalyst exhibited the lowest reduction temperature (137 °C). However, the proportion of the β reduction peak of 10CuO/NS-CeO_2_-500 catalysts was substantially higher than those of the 10CuO/NS-CeO_2_-300 and 10CuO/NS-CeO_2_-400 catalysts, as shown in Table 4. This suggested that the CuO active sites were still highly dispersed in catalysts calcined at high temperatures, which had been confirmed using the XRD analyses. Interestingly, despite the higher H_2_ reduction temperature of the 10CuO/NS-CeO_2_-500 catalyst compared to the 10CuO/NS-CeO_2_-300 and 10CuO/NS-CeO_2_-400 catalysts, it still exhibited the best CO oxidation activity. This indicated that the contents of Cu^+^ and lattice oxygen were the decisive factors determining the catalyst activity.

Figure 10c displays the H_2_-TPR profiles of the CuO-based catalysts with various supports. As could be observed, these four catalysts exhibited two or three reduction peaks. The H_2_ consumption peaks of the 10CuO/SiO_2_-500 catalyst at 291 °C and 342 °C were ascribed to the reduction in highly dispersed CuO on the SiO_2_ support and the reduction in agglomerated bulk CuO distributed on the SiO_2_ [66]. The H_2_ consumption peak of the 10CuO/Al_2_O_3_-500 catalyst at 243 °C was because of the reduction in highly dispersed CuO on the surface of Al_2_O_3_, and the reduction peak at 333 °C was because of the reduction in bulk CuO [67]. Notably, the reduction peak temperature of the 10CuO/NS-CeO_2_-500 catalyst was notably lower than those of the other three reference CuO-based catalysts with commercial supports. The mesoporous CeO_2_ nanosphere, as a reducible support, more easily participated in CO oxidation.

### 3.3. Catalytic Performances of CuO-Based Catalysts toward CO Oxidation

#### 3.3.1. Effect of the CuO Loading Amount on the Catalytic Activity of CO Oxidation

**Figure 11:**
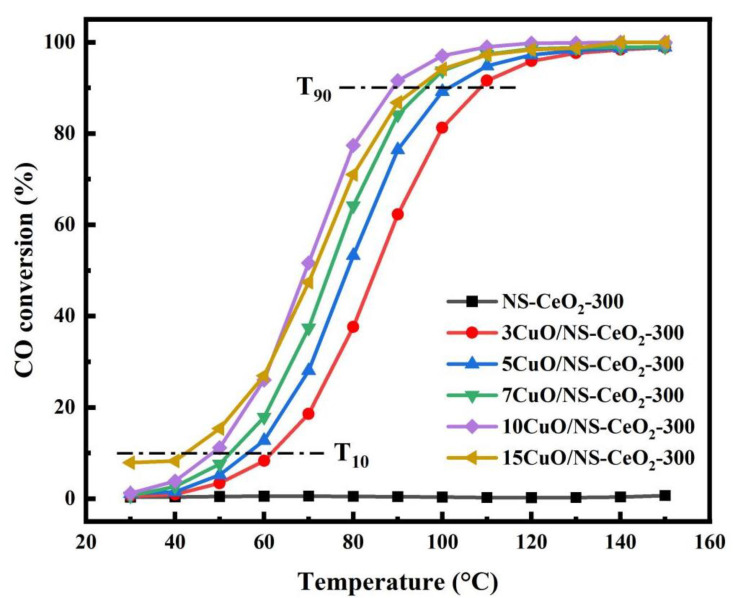
The curves of the CO conversion versus reaction temperature over the NS-CeO_2_-300 support and *x*CuO/NS-CeO_2_-300 catalysts; reaction conditions: CO/O_2_/N_2_ = 1/20/79, GHSV = 12,000 mL/(g·h), 1 atm.

The activities of the xCuO/NS-CeO_2_-300 catalysts toward CO oxidation were systematically evaluated at different reaction temperatures. It was found in Figure 11 that the loading amount of the CuO active site greatly influenced the activities of the xCuO/NS-CeO_2_-300 catalysts. The pure mesoporous CeO_2_ nanosphere support did not exhibit CO oxidation activity. The CO oxidation activity of the catalyst was usually evaluated and expressed in the form of the temperature (denoted as T_90_) at which the CO conversion reached 90% CO oxidation. Generally, the activities of the xCuO/NS-CeO_2_-300 catalysts gradually increased when the loading amount of the CuO increased from 3 wt.% to 10 wt.%. The reason for this was that the increase in the CuO loading amount increased the number of accessible CuO active sites for the gaseous reactants, accounting for the enhanced CO conversion. However, the catalytic activity decreased a bit when the content of CuO further increased to 15 wt.%, whereas extensively increasing the CuO loading amount up to 15 wt.% would cause the agglomeration of the active sites, causing a decrease in CO conversion. Specifically, the activities of the xCuO/NS-CeO_2_-300 catalysts expressed in the form of T_90_ observed the following order: 10CuO/NS-CeO_2_-300 (89 °C) > 15CuO/NS-CeO_2_-300 (94 °C) > 7CuO/NS-CeO_2_-300 (97 °C) > 5CuO/NS-CeO_2_-300 (100 °C) > 3CuO/NS-CeO_2_-300 (109 °C). Furthermore, the temperature, at which the CO conversion reached 10%, was defined as the catalyst activation temperature (denoted as T_10_). Slightly different from the order of T_90_, the activation temperature (T_10_) of the 15CuO/NS-CeO_2_-300 (42 °C) catalyst was lower than 10CuO/NS-CeO_2_-300 (48 °C). The activity of the 10CuO/NS-CeO_2_-300 catalyst was gradually higher than that of the 15CuO/NS-CeO_2_-300 catalyst when the conversion of CO increased from 10% to 90%. Therefore, the 10CuO/NS-CeO_2_-300 catalyst performed the best catalytic activity among these catalysts. Combined with the results from H_2_-TPR, this was due to the presence of highly dispersed CuO on the surface of the 10CuO/NS-CeO_2_-300 catalyst.

#### 3.3.2. Effect of the Calcination Temperature on the Catalytic Activities of CO Oxidation

**Figure 12:**
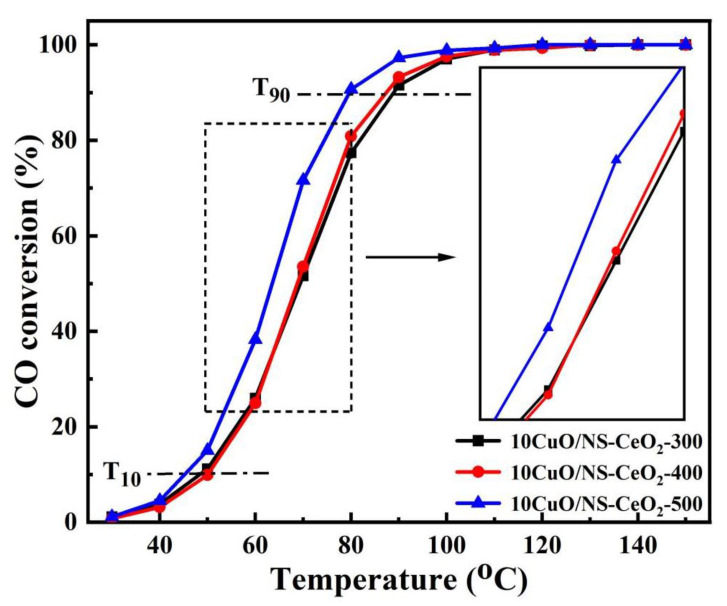
The curves of the CO conversion versus reaction temperature over the 10CuO/NS-CeO_2_-T catalysts; reaction conditions: CO/O_2_/N_2_ = 1/20/79, GHSV = 12,000 mL/(g·h), 1 atm.

The influence of the calcination temperature of the 10CuO/NS-CeO_2_-T catalysts for CO oxidation activities was studied. Figure 12 shows the CO conversions over the 10CuO/NS-CeO_2_-T catalysts. It was noticed that the catalytic activity increased with the increase in the calcination temperature. Specifically, the T_10_ of the 10CuO/NS-CeO_2_-500 catalyst (45 °C) was lower than those of the 10CuO/NS-CeO_2_-300 (49 °C) and 10CuO/NS-CeO_2_-400 catalysts (50 °C), demonstrating a lower activation temperature of the CO oxidation. Similarly, the T_90_ of the 10CuO/NS-CeO_2_-500 catalyst was also the smallest among these investigated catalysts. Specially, the sequence of T_90_ of these three 10CuO/NS-CeO_2_-T catalysts were listed as below: 10CuO/NS-CeO_2_-300 (89 °C) > 10CuO/NS-CeO_2_-400 (87 °C) > 10CuO/NS-CeO_2_-500 (80 °C). The 10CuO/NS-CeO_2_-500 catalyst displayed the highest activity. The possible reason was that the 10CuO/NS-CeO_2_-500 catalyst still possessed the large specific surface area, which would facilitate the effective dispersion of CuO active sites and further provide enough accessible CuO active sites for the gaseous reactants. Furthermore, it was found that the activities of the 10CuO/NS-CeO_2_-T catalysts were closely related to the Cu^+^ content and the lattice oxygen content of 10CuO/NS-CeO_2_-T catalysts, which also had a close relationship with the calcination temperature. Regarding the roles of oxygen species, such as lattice oxygen and the adsorbed oxygen, in the CO oxidation reaction over CuO/CeO_2_ catalysts, there has been no consensus so far in the previous reports. Liu et al. [68] proposed that surface-adsorbed oxygen played a major role in CO oxidation. However, Tang et al. [69] found that the lattice oxygen of the catalyst was involved in the oxidation of CO. Additionally, Zou et al. [70] reported that the reduction in both adsorbed oxygen and lattice oxygen might lead to a decrease in the activity of the catalyst. Their results indicated that both the adsorbed oxygen and lattice oxygen species could simultaneously participate in the CO catalytic oxidation reaction. In this work, it was found that the lattice oxygen was the main active oxygen species reacting with the CO reactant. Meanwhile, the 10CuO/NS-CeO_2_-500 catalyst also exhibited the highest Ce^3+^ content based on the XPS analysis. It was believed that the high concentration of Ce^3+^ facilitated the reduction in CuO and the generation of the Cu^+^ species, which served as the adsorption and active sites for the reactants during the CO oxidation [60,71]. The essential activity of the catalyst is typically evaluated using the specific surface area normalization [72]. The result of the specific surface area normalization reaction rate is summarized in Table 3. It can be observed that the results of reaction rates were consistent with the activity order of the catalyst. This indicated that the effect of the specific surface area on catalytic activity can be eliminated. The results of the normalized reaction rate further illustrated this, as well as confirming the positive effects of lattice oxygen content and Cu^+^ content on catalytic activity.

**Figure 13:**
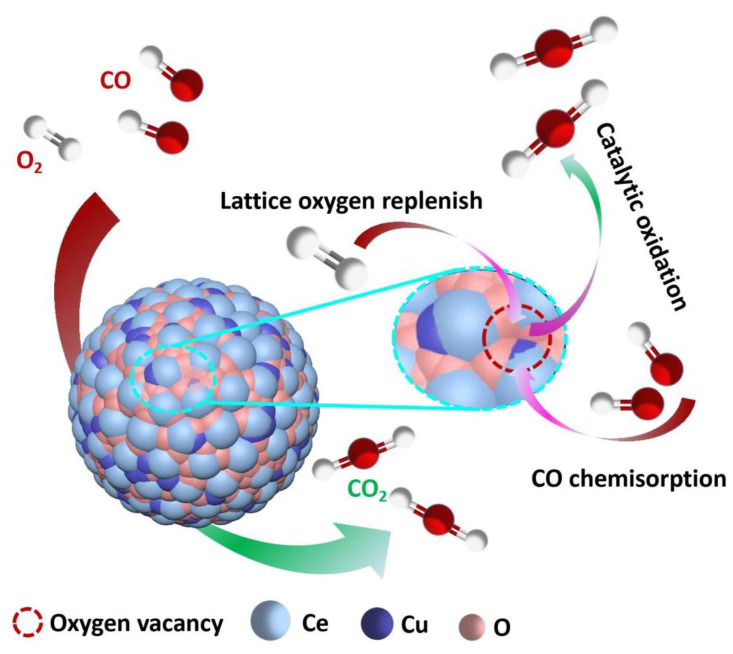
The reaction mechanism of the CO oxidation on the CuO supported catalyst supported on the mesoporous CeO_2_ nanosphere.

The proposed reaction mechanism of CO oxidation over the CuO/NS-CeO_2_ catalyst is illustrated in Figure 13. It was widely believed that the active sites for CO oxidation were located at the interface between CuO and CeO_2_ [61]. The redox electron pairs (Cu^2+^ + Ce^3+^ ↔ Cu^+^ + Ce^4+^) would be formed between CuO and CeO_2_, activating lattice oxygen in the vicinity of CuO in CeO_2_. The CO chemisorbed on Cu^+^ reacted with the activated oxygen species and/or lattice oxygen in CeO_2_. The gaseous oxygen species subsequently supplemented the reacted oxygen species and/or lattice oxygen on the oxygen vacancies [69,73]. It was proposed that the O_2_ was activated on the oxygen vacancies in CeO_2_ to regenerate the reactive oxygen species and/or lattice oxygen [73].

#### 3.3.3. Effects of Mesostructure and Redox Property of the Support on the Catalytic Activity of CO Oxidation

**Figure 14:**
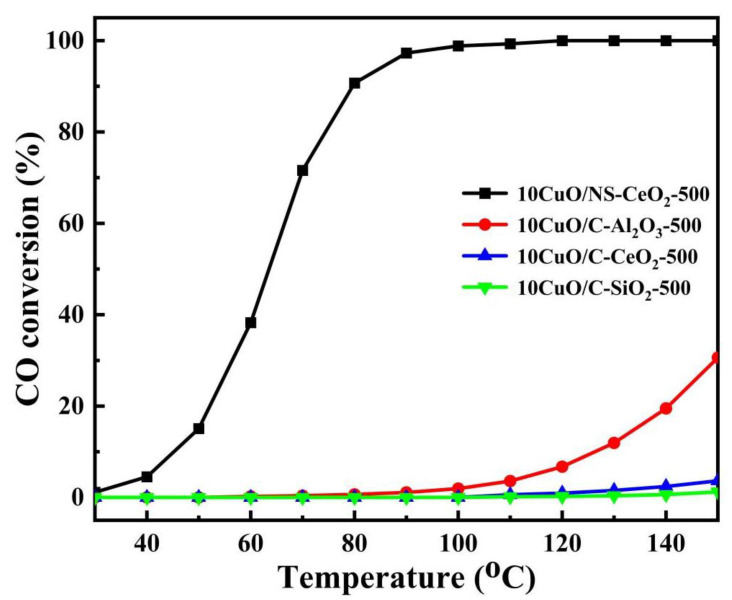
The curves of the CO conversion versus reaction temperature over the 10CuO/NS-CeO_2_-500, 10CuO/C-SiO_2_-500, 10CuO/C-Al_2_O_3_-500, and 10CuO/C-CeO_2_-500 catalysts; reaction conditions: CO/O_2_/N_2_ = 1/20/79, GHSV = 12,000 mL/(g·h), 1 atm.

To investigate the effects of the mesoporous structure and redox property of the support on the catalytic activity of CO oxidation; the 10CuO/C-SiO_2_-500, 10CuO/C-Al_2_O_3_-500 and 10CuO/C-CeO_2_-500 catalysts with commercial supports were used as the reference catalysts. The curves of the CO conversion versus reaction temperature over these catalysts are shown in Figure 14. As could be noticed, the 10CuO/C-SiO_2_-500 catalyst was basically not active toward CO oxidation even when the reaction temperature was up to 150 °C. The 10CuO/C-Al_2_O_3_-500 catalyst did not show any activity before 90 °C, and the CO conversion only reached 30.60% at 150 °C. Similar to this, the 10CuO/C-CeO_2_-500 catalyst was activated at 120 °C, and the CO conversion was achieved at 3.67% at 150 °C. In comparison, the CO conversion over the 10CuO/NS-CeO_2_-500 catalyst rapidly increased with the increase in temperature in the investigated temperature range (30–150 °C), and the CO conversion reached 100% at 120 °C. The activity of the 10CuO/NS-CeO_2_-500 catalyst was greatly enhanced compared to the activities of 10CuO/C-SiO_2_-500 and 10CuO/C-Al_2_O_3_-500. It was believed that the Cu^+^/Cu^2+^ and Ce^4+^/Ce^3+^ electron pairs were formed after loading the CuO active sites on the mesoporous CeO_2_ nanosphere. It was reported that the formation of Cu/Ce electron pairs (Cu^2+^ + Ce^3+^ ↔ Cu^+^ + Ce^4+^) could greatly increase the surface oxygen vacancies of the catalyst and the presence of more Ce^3+^ cations in the catalyst could evidently promote the formation of Cu^+^ in the catalyst [59,74]. The activity of the 10CuO/NS-CeO_2_-500 catalyst was also higher than the activity of the 10CuO/C-CeO_2_-500 catalyst. The morphology and specific surface area of the two CeO_2_ supports were different. The large specific surface area of NS-CeO_2_ support was conducive to the dispersion of CuO and further affected the valence and existence state of Cu species. As a result, the Cu^+^ content as well as the highly dispersible CuO content of the 10CuO/NS-CeO_2_-500 catalyst was much greater than that of the 10CuO/C-CeO_2_-500 catalyst. It was believed that the Cu^+^ in the CuO/CeO_2_ catalyst was beneficial to the adsorption of CO. Therefore, the CeO_2_ nanosphere with a large specific surface area was of great significance for increasing Cu^+^ active sites. Meanwhile, the 10CuO/NS-CeO_2_-500 catalyst also had the highest oxygen vacancy content. The oxygen vacancies in the 10CuO/NS-CeO_2_-500 catalyst increased the mobility of lattice oxygen in CeO_2_. This demonstrated the superiority of mesoporous CeO_2_ nanospheres with a high specific surface area.

Furthermore, the activities of CO oxidation over different CuO/CeO_2_ catalysts between this work and the literature were compared. The results are summarized in Table 5. It could be observed that the 10CuO/NS-CeO_2_ catalyst reported in work performed the lowest T_90_ temperature (80 °C) among the reported results, suggesting the highest catalytic activity. In this work, a large specific surface area CuO/NS-CeO_2_ catalyst with high CO oxidation activity was prepared using simple materials and methods. Compared with CuO/CeO_2_ catalysts obtained using different preparation methods, the preparation method of NS-CeO_2_ support is simpler. Compared with the CuO/CeO_2_ catalyst obtained using the impregnation method, the CO oxidation activity of the CuO/NS-CeO_2_ catalyst was higher. It was supposed that the large surface area of the NS-CeO_2_ support made a great contribution to the high catalytic activity of the 10CuO/NS-CeO_2_-500 catalyst.

#### 3.3.4. Stability Test of the 10CuO/NS-CeO_2_-500 Catalyst

**Figure 15:**
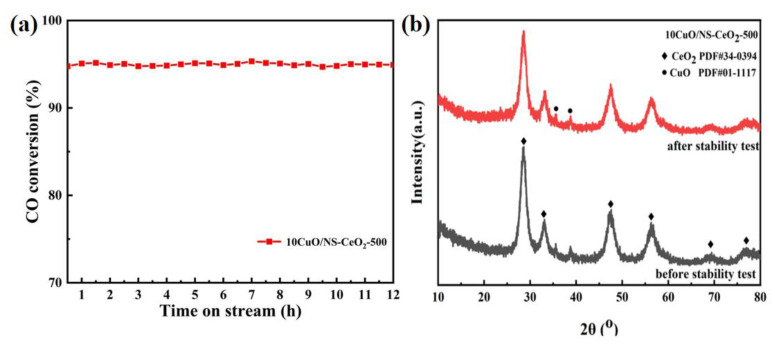
(**a**) The CO conversion versus time on stream over the 10CuO/NS-CeO_2_-500 catalyst; reaction condition: CO/O_2_/N_2_ = 1/20/79, GHSV = 12,000 mL/(g·h), 90 °C, 1 atm; (**b**) XRD patterns of the 10CuO/NS-CeO_2_-500 catalyst before and after 12 h long-term stability tests.

As the catalyst with outstanding activity, the 10CuO/NS-CeO_2_-500 catalyst was selected as the presentative for the 12 h catalyst stability test at 90 °C. The curve of the CO conversion versus time on the stream is shown in Figure 15a. It was observed that the 10CuO/NS-CeO_2_-500 catalyst displayed about 95% CO conversion and also did not show obvious deactivation during the 12 h stability test, demonstrating excellent catalytic stability.

The 10CuO/NS-CeO_2_-500 catalyst after the 12 h stability test of the CO oxidation reaction was characterized using the XRD to evaluate the effect of the stability test on the catalyst. Their XRD patterns are exhibited in Figure 15b. Compared with the XRD pattern of the 10CuO/NS-CeO_2_-500 catalyst before the stability test, the XRD pattern of the 10CuO/NS-CeO_2_-500 catalyst after the stability test did not exhibit any noticeable sintering or change in the crystalline phase. The results of the XRD characterization demonstrated that the 10CuO/NS-CeO_2_-500 catalyst was provided with excellent thermal sintering resistance.

## 4. Conclusions

In this work, the large specific surface area mesoporous CeO_2_ nanosphere with outstanding thermal stability was facilely synthesized using the facile hydrothermal method. The mesoporous CeO_2_ nanosphere was used as the support of the CuO-based catalysts for a CO oxidation reaction. The catalytic supports and the CuO-based catalysts were systematically characterized using various techniques, such as XRD, N_2_ physisorption, SEM, EDS-mapping, H_2_-TPR, XPS, in situ DRIFTS, etc. The effects of the CuO loading amount, catalyst calcination temperature, mesostructured support, and the redox property of the support on the catalytic activity of CO oxidation were also analyzed. It was observed that the catalyst with a mesoporous CeO_2_ nanosphere, 10 wt.% CuO loading amount, and 500 °C calcination temperature demonstrated the optimum CO oxidation activity with a T_90_ of 80 °C. This work also demonstrated that the mesoporous CeO_2_ nanosphere with a large specific surface area and redox property displayed much higher CO oxidation activity than the reference catalysts with commercial CeO_2_, SiO_2_, and Al_2_O_3_ supports. This was attributed to the large surface area of the mesoporous CeO_2_ nanosphere support, which was effective in highly dispersing CuO active sites even at high calcination temperatures. The result of the XPS analysis demonstrated that the 10CuO/NS-CeO_2_-500 catalyst with excellent redox properties performed the highest Cu^+^ and Ce^3+^ concentrations, which was advantageous for the chemisorption and activation of the gaseous reactants. Additionally, the 10CuO/NS-CeO_2_-500 catalyst also exhibited a high lattice oxygen content. These advantages accounted for the excellent performance of the 10CuO/NS-CeO_2_-500 catalyst toward CO oxidation. Therefore, the mesoporous CeO_2_ nanosphere with a large specific surface area was considered as an excellent catalyst to support the CuO-based catalysts for a CO oxidation reaction with enhanced performance.

## Figures and Tables

**Table 1:** Structural properties of the supports and catalysts based on the N_2_ physisorption analyses.

Catalysts	Specific Surface Area (m^2^/g)	Pore Volume (cm^3^/g)	Average Pore Diameter (nm)	Isotherm Type
NS-CeO_2_	190.4	0.18	3.4	IV H3
NS-CeO_2_-300	215.8	0.16	3.4	IV H3
NS-CeO_2_-400	208.4	0.17	3.4	IV H3
NS-CeO_2_-500	181.0	0.17	3.4	IV H3
C-CeO_2_	52.6	0.14	3.4	IV H3
C-Al_2_O_3_	170.3	0.27	3.8	IV H4
C-SiO_2_	251.1	0.71	3.4	IV H3
3CuO/NS-CeO_2_-300	201.9	0.20	3.5	IV H3
5CuO/NS-CeO_2_-300	194.9	0.18	3.4	IV H3
7CuO/NS- CeO_2_-300	183.1	0.19	3.4	IV H3
10CuO/NS-CeO_2_-300	164.2	0.16	3.4	IV H3
15CuO/NS-CeO_2_-300	137.0	0.14	3.4	IV H3
10CuO/NS-CeO_2_-400	131.5	0.14	3.4	IV H3
10CuO/NS-CeO_2_-500	176.0	0.18	3.4	IV H3
10CuO/C-CeO_2_-500	49.2	0.02	3.4	IV H3
10CuO/C-Al_2_O_3_-500	120.5	0.23	3.8	IV H4
10CuO/C-SiO_2_-500	215.9	1.18	31.0	IV H3

**Table 2:** Cu 2p peak areas and ratios of Cu^+^ over different catalysts derived from XPS analyses.

Catalysts	Cu^+^ Peak Area of Cu 2p _3/2_	(Cu^2+^ + Cu^+^) Peak Area of Cu 2p _3/2_	Cu^+^ Peak Area Ratio ^a^ (%)
10CuO/NS-CeO_2_-300	25,103.5	92,420.7	27.2
10CuO/NS-CeO_2_-400	26,307.4	91,707.9	28.7
10CuO/NS-CeO_2_-500	28,651.3	81,298.7	35.2
10CuO/C-CeO_2_-500	17,565.1	73,526.2	23.9
10CuO/C-Al_2_O_3_-500	7956.9	43,507.4	18.3
10CuO/C-SiO_2_-500	3954.8	14,713.7	26.9

^a^ The content of Cu^+^ can be calculated from the area ratio of Cu^+^/(Cu^2+^ + Cu^+^) × 100%.

**Table 3:** O 1 s peak areas, ratios of the lattice oxygen and surface adsorbed oxygen over the different catalysts, and the normalized reaction rate of 10CuO/NS-CeO_2_-T catalysts.

Catalysts	Lattice Oxygen Peak Area of O 1s (O_latt_)	Surface Adsorbed Oxygen Peak Area of O 1s (O_ads_)	O_ads_/(O_latt_ + O_ads_) (%)	O_latt_/(O_latt_ + O_ads_) (%)	The Normalized Reaction Rate ^a^ (10^−6^ mol·m^−2^s^−1^)
10CuO/NS-CeO_2_-300	110,631.7	100,654.3	47.64	52.36	3.55
10CuO/NS-CeO_2_-400	128,354.4	83,209.0	39.33	60.67	3.65
10CuO/NS-CeO_2_-500	132,391.4	67,324.0	33.71	66.29	3.91
10CuO/C-CeO_2_-500	118,402.2	54,983.0	31.71	68.29	0

^a^ Normalized reaction rates over the 10CuO/NS−CeO_2_−T (T = 300, 400, 500) catalysts under the reaction conditions: CO/O_2_/N_2_ = 1/20/79, GHSV = 12,000 mLg^−1^h^−1^, 40 °C, 1 atm.

**Table 4:** The quantitative data of the H_2_-TPR profiles of the as-prepared catalysts.

Catalysts	Temperature (°C)			Fraction of Total Area (%)		
	α	β	γ	α	β	γ
3CuO/NS-CeO_2_-300	197	—	224	42.7	—	57.3
5CuO/NS-CeO_2_-300	181	—	222	68.6	—	31.4
7CuO/NS-CeO_2_-300	179	—	229	62.7	—	37.3
10CuO/NS-CeO_2_-300	168	208	237	45.0	22.4	32.6
10CuO/NS-CeO_2_-400	137	176	215	13.0	31.0	56.0
10CuO/NS-CeO_2_-500	199	236	253	27.0	42.1	30.9
15CuO/NS-CeO_2_-300	220	—	254	57.0	—	43.0
10CuO/C-CeO_2_-500	349	—	407	66.8	—	33.2

**Table 5:** The activities of CO oxidation over different CuO/CeO_2_ catalysts.

Catalysts	Synthesis Methods	S_BET_ (m^2^/g)	T_90_ (°C)	References
CuO/CeO_2_-20	Solution combustion synthesis	52.7	110	[54]
30% CuO-CeO_2_	Hydrothermal method	102	100	[57]
10 wt% CuO/CeO_2_-NR	Wet impregnation method	112.9	195	[75]
6-CuO/CeO_2_	Wet impregnation method	55.4	254	[64]
Cu/Ce(SI)	Surfactant-assisted impregnation	9.7	92	[71]
5Cu/CeO_2_(IM)	Impregnation method	76.3	108	[76]
550-CC (Cu: Ce molar ratio 1:4, activation in air at 550 °C)	Facile one-step solvothermal synthesis	62	87	[77]
N8A4-10 (CuO-CeO catalyst treated in N_2_ at 800 °C and in air at 400 °C, and the CuO content in the catalyst is 5 mol %)	Modified citrate sol–gel method	131	100	[32]
10 wt.% Cu (CP)	Coprecipitation method	117.6	85	[23]
10CuO/NS-CeO_2_-500	Incipient impregnation method	176	80	This work

## Data Availability

The data presented in this study are not available due to privacy.

## Supplementary Materials

The following supporting information can be downloaded at https://www.mdpi.com/article/10.3390/nano14060485/s1. Table S1. Ce 3d peak area of the catalysts based on XPS analysis; Table S2. Binding energies (eV) of the surface Cu 1s, O 1s, and Ce 3d elements of the as-prepared catalysts. Reference [72] is cited in the Supplementary Materials.
