# Supplementary material for: Constructing Efficient CuO-Based CO Oxidation Catalysts with Large Specific Surface Area Mesoporous CeO2 Nanosphere Support
Source: Nanomaterials (Basel). 2024 Mar 7;14(6):485. doi: 10.3390/nano14060485 (PMC10974734; doi:10.3390/nano14060485)
Supplement: Supplementary file 1 [file nanomaterials-14-00485-s001.zip › nanomaterials-2894642-supplementary.pdf]

nanomaterials

MDPI

Supplementary Materials

Constructing Efficient CuO-Based CO Oxidation Catalysts with Large Specific Surface Area Mesoporous CeO_2 Nanosphere, Support

Yixin Zhang [1,†], Fen Zhao [1,†], Hui Yang [1], Siyuan Yin [1], Cai-E Wu [2], Tingting Zhou [3], Jingxin Xu [4], Leilei Xu [1,*] and Mindong Chen [1,5,*]

[1] Collaborative Innovation Centre of the Atmospheric Environment and Equipment Technology, School of Environmental Science and Engineering, Nanjing University of Information Science & Technology, Jiangsu Key Laboratory of Atmospheric Environment Monitoring and Pollution Control, Joint International Research Laboratory of Climate and Environment Change (ILCEC), Nanjing 210044, China; 20211212034@nuist.edu.cn (Y.Z.); 20201248169@nuist.edu.cn (F.Z.); 20211248130@nuist.edu.cn (H.Y.); 20211248096@nuist.edu.cn (S.Y.)

[2] College of Light Industry and Food Engineering, Nanjing Forestry University, Nanjing 210037, China; wucaie@njfu.edu.cn

[3] College of Chemical Engineering and Environmental Chemistry, Weifang University, Weifang 261061, China; 20170023@wfu.edu.cn

[4] State Key Laboratory of Low-Carbon Smart Coal-Fired Power Generation and Ultra-Clean Emission, China Energy Science and Technology Research Institute Co., Ltd., Nanjing 210023, China; 20060331@ceic.com

[5] School of Environment and Energy Engineering, Anhui Jianzhu University, Hefei 230009, China

* Correspondence: leileixu88@gmail.com (L.X.); chenmd@nuist.edu.cn (M.C.)

† These authors contributed equally to this work.

S1. The synthesis of the mesoporous CeO_2 nanosphere

In a specific preparation process, 1.0 g $Ce(NO_3)_3·6H_2O$ (Shanghai Macklin Bio-Chem Co., Ltd., China) was dissolved in 1.0 mL deionized water, then 30.0 mL glycol (Sinopharm Chemical Reagent Co., Ltd., China) and 1 mL acetic acid (Shanghai Meryer Co., Ltd., China) were added with vigorous agitation at room temperature for 15 min. After the stirring, the mixed solution was transferred to the steel autoclave lined with Teflon and kept in the 180 °C oven for 150 min hydrothermal reaction. After the reaction was completed, the steel autoclave was naturally cooled to room temperature. The powder was separated by the centrifugation operation and washed alternately with deionized water and absolute ethanol (C_2H_5OH, Sinopharm Chemical Reagent Co., Ltd., China) two times. Finally, the powder was dried in the vacuum drying oven at 60 °C overnight. The finally obtained mesoporous CeO_2 nanosphere was labeled as the NS-CeO_2-P, where the P represented the precursor.

S2. The preparation of the CuO/NS-CeO_2 supported catalysts

The CuO-based catalyst with x wt.% CuO (x wt.% = $m_{CuO}/(m_{CuO} + m_{support})$) loading amount was synthesized by the incipient impregnation method with the NS-CeO_2-P as support and the $Cu(NO_3)_2·3H_2O$ (Sinopharm Chemical Reagent Co., Ltd., China) as precursor. After the impregnation process, the catalyst precursor was dried in 70 °C oven overnight, and then was calcined at different temperature for 4 h with the heating rate of 0.5 °C/min. The as-prepared catalysts were labeled as *x*CuO/NS-CeO_2-T, where *x* represented the mass fraction of the CuO active sites and T represented the calcination temperature.

S3. Catalyst characterizations

The powder X-ray diffraction (XRD) patterns were recorded on the XRD-6100 diffractometer (Shimazu, Japan) using the Cu Kα (40 kV, 100 mA) radiation in the scanning range of 20-80 ^{o} (2θ) with the scanning rate of 10 ^{o} /min.

The N_2 physisorption was performed on the Autosorb-IQ-Ag-MP instrument (Quantachrome, USA) at liquid nitrogen temperature (-196 °C). Prior to analysis, the sample was degassed at 300 °C for 3 h to remove the surface adsorbed water and impurities. The specific surface area of the catalyst was calculated by the multipoint Brunauer-Emmett-Teller (BET) method in the range of 0-1.0 P/P_0 and the pore size distribution and pore volume were calculated by the Barrett-Joyner-Halenda (BJH) method from the adsorption branch of the isotherm.

Scanning electron microscope (SEM) images were taken on the Apreo S Hivac instrument (Thermo Fisher Scientific, USA) with 5 kV acceleration voltage.

Transmission electron microscope (TEM) images were taken on the JEOL JEM-2100Plus instrument (JAPAN).

The *in-situ* diffused reflectance infrared Fourier transform spectroscopy (*in-situ* DRIFTS) spectra of the catalyst calcination processes were collected by using the Nicolet IS 500 FT-IR spectrum spectrometers (Thermo Fisher Scientific, USA) equipped with a modified Harrick Praying Mantis DRIFT cell. The signal of the functional groups of the samples during the calcination process was measured. Before the analysis, the catalyst precursors were ground uniformly and pretreated at 60 °C in a vacuum oven for 12 h. The pretreated sample (0.5 g) was put into the cell. The temperature of the cell loaded with sample was then increased from 35 °C to 500 °C with the ramping rate of 10 °C /min.

X-ray photoelectron spectroscopy (XPS) measurements were conducted on the Thermo Scientific K-Alpha+ spectrometer (Thermo Fisher Scientific, USA). The binding energies of the XPS spectra were calibrated with the C 1s binding energy of 284.5 eV as the reference.

The H_2 temperature programmed reduction (H_2-TPR) was measured on the home-made fixed bed reactor and the H_2 consumption profile was recorded by the online LC-D200 mass spectrometer (TILON GRP TECHNOLOGY LIMITED, USA). For each test, 50 mg of catalyst was loaded and the mixture of H_2 (0.4 mL/min) and Ar (7.6 mL/min) was introduced into the reactor. After the H_2 signal baseline (m/z = 2) was stable, the H_2-TPR operation was performed from the 30 °C to 800 °C with the heating rate of 20 °C /min.

S4. Catalytic activity measurements

The performance of the catalyst for CO oxidation was evaluated on a vertical fixed-bed continuous flow reactor equipped with quartz tube (i.d. = 8.0 mm). The flow rates of the reactant gases were regulated by the mass flow controllers. The reaction temperature was monitored and precisely controlled by two thermocouples located in the center of the fixed-bed reactor, respectively. For each test, 0.1 g catalyst was loaded in the center of the quartz tube supported by the quartz wool. The feed gas was consisted of 1.0 vol% CO, 20.0 vol% O_2, and 79.0 vol% N_2. The total flow rate was 20.0 mL/min and the corresponding gas hourly space velocity (GHSV) was 12,000 mL/(g·h). The catalytic activities of CO oxidation on different catalysts were evaluated in the specific temperature range of 30-150 °C. The outlet gas from the fixed-bed reactor was analyzed by the online Techcomp GC-7900 gas chromatograph equipped with the automatic six-way injection valves. The activity of the catalyst was expressed in the form of CO conversion rate. Moreover, considering the carbon balance, the CO conversion rate (abbreviated as X_{CO}) could be calculated based on the following formula, where $F_{CO,\ inlet}$ and $F_{CO,\ outlet}$ (ppm) were the flows of CO species flowing into and out of the reactor, respectively.

$$X_{CO}\ (\%) = \frac{F_{CO,\ inlet} - F_{CO,\ outlet}}{F_{CO,\ inlet}} \times 100\% \quad (1)$$

S5. The calculation of the normalized reaction rate

The calculation of the normalized reaction rate (r_{norm}, $mol \cdot m^{-2} \cdot s^{-1}$) was derived from the following formula reported in the pioneer literature [1]:

$$r_{norm}\ (mol \cdot m^{-1} \cdot s^{-2}) = \frac{C_{inlet} \cdot F}{m_{cat} \cdot S_{BET}} \cdot \ln\left(\frac{1}{1\text{-}X_{CO}}\right) \quad (2)$$

Where C_{inlet} is the concentration of CO in inlet gas, F ($mol \cdot s^{-1}$) is the CO flow rate, m_{cat} (g) is the mass of catalyst, S_{BET} ($m^2 \cdot g^{-1}$) is the BET surface area, the X_{CO} is the CO conversion at 40 °C.

Table S1. Ce 3d peak area of the catalysts based on XPS analysis.

**Catalysts**	**Ce^{3+} peak area of Ce 3d**	**Ce^{3+} + Ce^{4+} peak area of Ce 3d**	**Ce^{3+} peak area ratio[a] (%)**
10CuO/NS-CeO_2-300	235221.4	1335285.3	17.62
10CuO/NS-CeO_2-400	188314.5	1406122.9	13.39
10CuO/NS-CeO_2-500	264884.8	1419829.5	18.66
10CuO/C-CeO_2-500	124421.4	793761.1	15.67

[a] Ce^{3+} could be calculated from the area ratio of peak (v'+ u')/(u + v + u' + v' + u'' + v''+ u''' + v''') × 100%.

Table S2. Binding energies (eV) of the surface Cu 1s, O 1s, and Ce 3d elements of the as-prepared catalysts.

**Catalysts**	**Cu $2p_{3/2}$**	**O 1s**	**Ce $3d_{5/2}$**
10CuO/NS-CeO_2-300	933.7	529.2	882.7
10CuO/NS-CeO_2-400	933.4	529.1	882.4
10CuO/NS-CeO_2-500	933.3	529.0	882.5
10CuO/C-CeO_2-500	933.6	529.1	882.3
10CuO/C-Al_2O_3-500	933.0	—	—
10CuO/C-SiO_2-500	933.4	—	—

References

1. Yang, W.; Su, Z.; Xu, Z.; Yang, W.; Peng, Y.; Li, J. Comparative study of α-, β-, γ-and δ-MnO_2 on toluene oxidation: Oxygen vacancies and reaction intermediates. *Appl. Catal. B Environ.* **2020**, *260*, 118150.
